# Childhood ADHD and Executive Functioning: Unique Predictions of Early Adolescent Depression

**DOI:** 10.1007/s10802-021-00845-6

**Published:** 2021-12-04

**Authors:** Michelle C. Fenesy, Steve S. Lee

**Affiliations:** 1 Department of Child & Adolescent Psychiatry, New York-Presbyterian Hospital, Columbia University Irving Medical Center, 622 W. 168th St., New York, NY 10032 USA; 2 Department of Psychology, University of California, Box 951563, 1285 Franz Hall, Los Angeles, CA 90095-1563 USA

**Keywords:** ADHD, Executive functioning, Depression, SEM

## Abstract

Given the increasing prevalence of adolescent depression, identification of its early predictors and elucidation of the mechanisms underlying its individual differences is imperative. Controlling for baseline executive functioning (EF), we tested separate ADHD dimensions (i.e., inattention, hyperactivity-impulsivity) as independent predictors of early adolescent depression, including temporally-ordered causal mediation by academic functioning and social problems, using structural equation modeling. At baseline, participants consisted of 216 children (67% male) ages 6–9 years old with (*n* = 112) and without (*n* = 104) ADHD who subsequently completed Wave 2 and 3 follow-ups approximately two and four years later, respectively. Predictors consisted of separate parent and teacher ratings of childhood ADHD and laboratory-based assessments of key EF domains. At Wave 2, parents and teachers completed normed rating scales of youth academic and social functioning; youth completed standardized assessments of academic achievement. At Wave 3, youth self-reported depression. Baseline inattention positively predicted early adolescent depression whereas childhood hyperactivity-impulsivity and EF did not. Neither academic nor social functioning significantly mediated predictions of depression from baseline ADHD and EF. We consider prediction of early adolescent depression from inattention, including directions for future intervention and prevention research.

In the United States, the prevalence of major depressive disorder has risen consistently since 1940 and currently incurs over $210 billion annually, a 21% increase since 2005 (Greenberg et al., 2015). The increase in prevalence coincides with an earlier age of onset (Birmaher & Brent, 2007): 2% of children and up to 8% of adolescents meet diagnostic criteria for major depressive disorder with an additional 5–10% of youth experiencing subclinical symptoms. Crucially, 30% of youth with major depressive disorder experienced suicidal ideation in the past 12 months (Avenevoli et al., 2015). Moreover, depression and suicidality have increased precipitously since 2010 (Twenge et al., 2017), including suicide being the second leading cause of death among 10–24-year-old individuals (Center for Disease Control and Prevention, 2016). To reduce its considerable clinical and public health burden, characterizing predictors of early adolescent depression is a priority.

Although understudied relative to co-occurring externalizing problems, cross-sectional associations of ADHD with depression (i.e., heterotypic comorbidity) are well-established (Humphreys et al., 2013; Meinzer et al., 2014; Seymour et al., 2014). Further, even with control of demographic and clinical factors (e.g., maternal depression), childhood and adolescent ADHD each uniquely predict depression through young adulthood (Chronis-Tuscano et al., 2010; Meinzer et al., 2016), and children with ADHD frequently experience recurrent depressive episodes (Chronis-Tuscano et al., 2010). ADHD is optimally conceptualized as the quantitative extreme of inattention and hyperactivity-impulsivity, but ADHD is typically examined dichotomously, thereby preventing specific inferences about the relative association of inattention and hyperactivity-impulsivity with respect to depression. In key exceptions, inattention—not hyperactivity-impulsivity—was uniquely associated with depression in 5–10-year-old youth (Fenesy & Lee, 2019; Humphreys et al., 2013), although hyperactivity-impulsivity predicted depression indirectly via emotion regulation (Seymour et al., 2014). In addition to the independent prediction of depression from separable dimensions of ADHD, there is a pressing need to identify additional factors exacerbating or mitigating risk.

Several lines of evidence suggest the plausibility of executive functioning (EF) as a risk factor with respect to youth depression. Although a precise definition of EF is somewhat intractable (Barkley, 2012), there is general consensus that EF encapsulates related cognitive processes (i.e., inhibitory control, working memory, set shifting) associated with the prefrontal cortex that support goal-directed behaviors (Barkley, 2012; Miyake et al., 2000; Nigg, 2017). Importantly, individuals with depression consistently exhibit poor emotion regulation, which is directly supported by EF among adolescents and adults (Gyurak et al., 2012; Joormann & Gotlib, 2010; Wagner et al., 2015). Further, emotion regulation tasks are sensitive to differential patterns of activation in prefrontal cortex regions associated with EF that suppress emotional responses from the limbic system (Zelazo & Cunningham, 2007). Moreover, rumination (i.e., engaging in repetitive thinking patterns that amplify emotions) is central to the onset and maintenance of depression (McLaughlin & Nolen-Hoeksema, 2011); elevated rumination mediated the cross-sectional association between impaired EF (i.e., set shifting) and depressive symptoms in typically developing adolescents (Dickson et al., 2017). Finally, individuals with poor EF often experience difficulties with goal attainment and planning across domains, thus catalyzing stress generation and potentially subsequent depression (Snyder & Hankin, 2016). Overall, there is growing evidence that EF is correlated with youth depression, although findings are frequently based on cross-sectional designs (e.g., Favre et al., 2008) and consist of broad age ranges (e.g., childhood through adolescence), preventing specific inferences about EF as a correlate versus risk factor for early-adolescent depression. Further, separable EF dimensions require rigorous measurement strategies to adequately capture its multidimensionality. Despite their empirical separability, boundaries among EF dimensions may be less distinct in children, giving rise to a unified set of cognitive processes (Lee et al., 2013; Wiebe et al., 2011), thus necessitating careful methodological and developmental considerations. Indeed, when derived from a single latent variable, poor preschool EF prospectively predicted early school-age depression symptoms, controlling for baseline depression (Nelson et al., 2018). However, this study did not extend into early adolescence, an important limitation given the precipitous increase in depression secondary to pubertal onset (Avenevoli et al., 2015). There is a pressing need to consider the *prospective* association of EF with depression during the transition from childhood to *early* adolescence using latent variable approaches. Specifically, this evidence will inform the potential need/utility of EF and/or behavioral interventions prior to depression onset in adolescence.

To clarify whether ADHD and EF *independently* predict youth depression, ADHD and EF must be examined concurrently. ADHD principally reflects executive dysfunction (Barkley, 1997; Castellanos & Tannock, 2002) with meta-analytic evidence that youth with ADHD exhibit impaired EF in community-based and clinical samples (Willcutt et al., 2005). Although EF is commonly proposed as a cognitive endophenotype or risk factor for ADHD (Gau & Shang, 2010; Nigg et al., 2004), controversy remains as there is considerable heterogeneity in EF among individuals with ADHD and not all children with a diagnosis of ADHD exhibit EF deficits (Kofler et al., 2019; Willcutt, et al., 2005). Further, the broader field of EF research seeks to clarify whether individual differences in EF represent a risk-factor for psychopathology, a correlate of psychopathology, or a result of psychopathology across multiple disorders (Snyder et al., 2015). Therefore, it is reasonable to evaluate EF as a correlate of ADHD. The present study aims to test the prospective prediction of depression from ADHD *and* EF, as controlling for comorbidities is recommended when evaluating EF in relation to depressive symptoms (McClintock et al., 2010).

Relatively few studies simultaneously examined the association of ADHD and EF with youth depression. For example, working memory, a key dimension of EF, was impaired among adolescents with ADHD + depression relative to youth with depression alone (Roy et al., 2017), but depression and ADHD were exclusively youth self-reported, despite the utility of multiple informants (Martel et al., 2017). Moreover, controlling for inattention and hyperactivity-impulsivity, EF dimensions (i.e., working memory, mental flexibility, inhibition) were unrelated to parent- or self-reported depression among 9–16-year-old youth (Øie et al., 2016). However, latent variable approaches improve measurement error; latent EF was positively associated with parent-rated depression among 5–10-year-old children (Fenesy & Lee, 2019). Among hospitalized inpatients, youth with depression and EF deficits had elevated ADHD compared to children with depression alone (Weber et al., 2018), although directional inferences were unclear, further underscoring the need for multi-method/informant longitudinal studies.

In addition to examining the independent prediction of depression from childhood ADHD and EF, it is necessary to investigate ADHD diagnostic status as a moderator of the prospective association between childhood EF and early adolescent depression to evaluate possible interactive effects. ADHD and EF interact to predict other key outcomes such as academic achievement. For example, elevated inattentive symptoms and poor EF predicted special education (Diamantopoulou et al., 2007). Thus, inclusion of ADHD diagnostic status as a moderator will reveal whether the prospective association between EF and depression varies according to ADHD diagnostic status. Testing independent and interactive prediction of depression from ADHD and EF will elucidate their contributions, informing how specific assessment and intervention approaches must be tailored.

Beyond predictive models, innovations in intervention require elucidation of underlying mechanisms of predictions of psychopathology from EF and ADHD (Meinzer & Chronis-Tuscano, 2017; Snyder et al., 2015; Zhou et al., 2012), a critical next step in EF research (Snyder et al., 2019). Both dual failure and competency-based models propose academic and social difficulties as key factors in the development of youth depression (Cole, 1991; Patterson & Stoolmiller, 1991). Whereas the dual failure model hypothesized that academic failure and peer rejection mediated predictions of depression from early conduct problems (McCarty et al., 2008; Patterson & Stoolmiller, 1991), competency-based models contend that children internalize negative feedback from the environment, which adversely affects their self-esteem and increases vulnerability to depression (Harter & Marold, 1994). Additionally, ADHD and EF each predicted academic achievement (Best et al., 2011; Miller et al., 2012) and social functioning (Diamantopoulou et al., 2007; Huang-Pollock et al., 2009), representing plausible mediators of depression from ADHD and EF. In particular, these theoretically-justified mediators should be subjected to stringent tests of *causal mediation* wherein constructs are temporally ordered relative to predictors and outcomes (MacKinnon & Fairchild, 2009).

Overall, the extant literature does not consider separable dimensions of ADHD in conjunction with EF, and prospective studies are necessary to determine whether EF represents a predictor versus correlate of early adolescent depression. Lastly, identification of underlying mechanisms is necessary to identify targets for intervention to reduce the risk of youth depression. To address these significant limitations hindering innovations in understanding the antecedents and mediators of early adolescent depression, the present study employed structural equation modeling (SEM) to evaluate inattention, hyperactivity-impulsivity, and EF as independent, prospective predictors of early adolescent depression controlling for key covariates (see Data Analytic Procedure). Additionally, we tested ADHD diagnostic status as a moderator to examine whether the prospective prediction of depression from EF differs for children with and without ADHD. Determining whether the prospective association between EF and depression varries according to group status would inform clinicans for whom the screening of EF is most important when evaluating risk for depression. We also tested whether academic and social functioning temporally mediated predictions from inattention, hyperactivity-impulsivity, and EF. Consistent with recommendations for SEM, if initial models did not fit the data, we thoughtfully respecified the models (Weston & Gore Jr., 2006; Violato & Hecker, 2007.) We hypothesized that inattention would positively predict whereas EF would inversely predict early adolescent depression. Lastly, we expected that academic and social functioning would each significantly mediate predictions of adolescent depression from inattention, hyperactivity-impulsivity, and EF.

## Methods

### Participants

Participants were 216 children (67% male) with (*n* = 112) and without (*n* = 104) ADHD and their families. Drawn from a large metropolitan city in the Western U.S., children were recruited from referrals from pediatric offices, mental health service agencies, and local schools. Inclusion criteria were English fluency and living with a biological caregiver at least halftime; exclusion criteria consisted of an IQ below 70 or seizure, autism spectrum, or other neurological condition. Table 1 summarizes key demographic data for participants. The sample was ethnically diverse (51.93% Caucasian; 8.33% African American; 12.04% Hispanic; 3.70% Asian; 22.22% Mixed; 1.85% Other/Unknown) and 29.95% of families had an annual income of $70,000 or less. At baseline (i.e., Wave 1), participating children were 6- to 9-years-old (*M* = 7.39, *SD* = 1.07). Families were invited to a follow-up approximately two years later (i.e., Wave 2) at which point youth ranged in age from 7- to 13- years old (*M* = 9.68, *SD* = 1.27). A final follow-up (i.e., Wave 3) was conducted about two years after Wave 2 when they were 9- to 15-years old (*M* = 12.07, *SD* = 1.30). Data from all three waves were utilized in the current study.

Of the 216 Wave 1 participants, 89.35% (n = 193) and 80.10% (n = 173) participated in the Wave 2 and 3 assessment, respectively. There were no significant differences in child age, sex, race, or baseline ADHD symptoms between children who participated at Wave 1 and Wave 2 or Wave 1 and Wave 3 (*p*s > 0.15). Youth who participated at Waves 2 and/or 3 had a higher mean IQ (*p*s < 0.01) than those who did not complete follow-ups. Overall, missing data ranged from approximately 72% on a youth self-rated depression measure to 0% on baseline ADHD data from a structured diagnostic interview. Maximum likelihood estimation addressed missing data (described further below).

### Procedures

Families initially completed a phone screen to determine eligibility. Parents and teachers of eligible participants were mailed rating scales of child functioning and were invited to a lab-based assessment conducted by well-trained graduate students in clinical psychology or B.A. level staff. After obtaining parental consent and youth assent, parents completed structured diagnostic interviews and rating scales to assess youth social-emotional functioning, whereas youth completed standardized tests of cognition, EF, and academic achievement as well as measures of social-emotional functioning. Researchers requested that children complete a medication washout for the day of testing, however this was optional. A total of 32 children with ADHD at baseline were prescribed medication, and 13 of these children took a simulant medication on the day of Wave 1 testing (6.02% of the total sample; *N* = 216). Wave 2 and Wave 3 follow-up assessments consisted of parallel procedures to assess similar constructs. The University of California, Los Angeles IRB approved all study procedures.

## Measures

### Wave 1 Predictors

***ADHD.*** We administered the ADHD module of the Diagnostic Interview Schedule for Children (DISC-IV) Parent Edition (Shaffer et al., 2000) to parents to assess Diagnostic and Statistical Manual of Mental Disorders DSM-IV ADHD. The DISC-IV is a computer-assisted, structured interview of symptoms, onset, and impairment with strong psychometric properties. The ADHD module has high internal consistency (*α* = 0.84; Schaffer et al., 2000). Given its superior predictive validity (Fergusson & Horwood, 1995), we analyzed the number of inattention (0–9) and hyperactivity-impulsivity symptoms (0–9) from the DISC-IV. Parents and teachers also rated ADHD symptoms on the Disruptive Behavior Disorder Rating Scale (DBD). Responses ranged from 0 (*not at all*) to 3 (*very much*; Pelham et al., 1992), yielding separate inattention and hyperactivity-impulsivity totals (0–27). The inattention and hyperactivity-impulsivity scales demonstrated high reliability within the present sample (parent-rated inattention *α* = 0.94; teacher-rated inattention *α* = 0.93; parent-rated hyperactivity-impulsivity *α* = 0.92; teacher-rated hyperactivity-impulsivity *α* = 0.94). Raw scores were used for inattention and hyperactivity-impulsivity variables as adjusted norms are not provided for these measures.

***Executive Functioning (EF***). For set shifting, we administered the child version of the Trail Making Test (Reitan & Wolfson, 1992). On Part A, participants drew lines as quickly as possible to sequentially connect numbered circles from 1 to 15 without making errors. Part B (TMT-B) involves alphabetically and numerically alternating between connecting numbers 1 through 13 and letters A through L. The time (*min*) to complete TMT-B reflects set shifting, with longer completion times indicating worse EF (Reitan & Wolfson, 1992). TMT-B significantly differentiated youth with ADHD and controls (Martel et al., 2007) and loaded onto a latent set shifting factor in children (Arán Filippetti & Richaud, 2017). To aid in interpretation, completion time was reverse coded so that higher scores represented better EF across all tests.

Participants completed the Children’s Version of the Golden Stroop (Golden et al., 2003) to assess inhibitory control. In the first condition, participants read as many words (i.e., red, blue, green) as possible in 45 s. The second condition required naming different colors of ink (i.e., red, blue, green) in the same 45 s timeframe. In the third and final condition (i.e., Color-Word [C-W]), the color names are printed in discordant colors. The total score on the C-W condition is the number of ink colors named in 45 s. The Stroop C-W shows strong criterion validity in children and adults (Arán Filippetti & Richaud, 2017; Miyake et al., 2000). The raw C-W score was used to estimate inhibitory control.

Children completed the Digit Span subtest from the Wechsler Intelligence Scale for Children-IV (Wechsler, 2003). In the forward condition, the examiner presents a string of numbers aloud; participants must recall the numbers in the proper order. The backward condition requires recalling the numbers in the reverse order. The Digit Span Backwards (DSB) raw score was used to estimate working memory given that it loaded more strongly on a latent working memory factor of EF relative to the forward condition (Arán Filippetti & Richaud, 2017). Normed scores do not exist for the raw backwards portion of the Digit Span subtest. Therefore, raw scores were utilized for all EF measures and we controlled for age on the latent EF variable.

### Wave 2 Mediators

***Academic Functioning***. Youth completed the Word Reading and Math Reasoning subtests from the Wechsler Individual Achievement Test Second Edition (WIAT-II; Wechsler, 2002), which assessed phonological awareness/decoding and mathematical problem solving, respectively. Separate Word Reading and Math Reasoning standard scores were employed.

Parents and teachers completed the Child Behavior Checklist (CBCL) and Teacher Report Form (TRF), respectively (Achenbach & Rescorla, 2001). The CBCL and TRF consist of 113-items with behaviors rated from 0 (*not true*) to 2 (*very true/often true*). The CBCL includes School Competence items, ranging from 0–6, to assess grades, class placement, grade repetition, and other problems in the school setting. Items from the school competence scale differentiate between youth from clinic referred and non-referred samples and has an acceptable reliability according to the manual (*α* = 0.63; Achenbach & Rescorla, 2001). The TRF Academic Performance scale is the mean of the child’s performance across academic subjects with a test–retest reliability of *r* = 0.90 (Achenbach & Rescorla, 2001). We used the CBCL School Competence and the TRF Academic Performance T-scores to create a latent academic functioning variable. All four observed variables for the proposed academic functioning latent factor provided adjusted values; thus, T-scores were utilized.

***Social Problems***. Parents and teachers completed the Dishion Social Preference Scale, a three-item (5-point metric) measure of peer acceptance, rejection, and being ignored (Dishion, 1990). Negative social preference was calculated by subtracting the reject from the accept rating and then reversing scoring the difference (Humphreys et al., 2013; Lee & Hinshaw, 2006). In addition to the academic functioning scales, the CBCL and TRF yield parallel Social Problems scales, which are reliable (*α* = 0.82 for both) and valid (Achenbach & Rescorla, 2001). These two variables were coupled with negative social preference to collectively estimate social problems. Because the Dishion Social Preference Scale does not provide age and sex adjusted norms, we used the raw scores for all four social variables for consistency across this proposed latent variable.

### Wave 3 Youth Self-Report Outcomes

**Depression*.*** Youth completed the 27-item Children’s Depression Inventory (CDI; Kovacs, 1992), rating descriptions from the past two weeks (e.g., “I feel like crying every day,” “I feel like crying many days,” “I feel like crying once in a while”). Each item is scored from 0 to 2, providing age- and sex-adjusted T-scores. The CDI has shown strong convergent validity with internalizing and disruptive behavior (Timbremont et al., 2004). The CDI demonstrated acceptable reliability within our sample (*α* = 0.85).

The 47-item Revised Children’s Anxiety and Depression Scale (RCADS) includes five normed subscales (Chorpita et al., 2000). Items were rated from 0 to 3, reflecting *never*, *sometimes*, *often*, or *always*, respectively. The sex- and grade-adjusted T-score from the Major Depressive Disorder subscale, which previously correlated with the CDI (Chorpita et al., 2000) and shows satisfactory internal consistency within this sample (*α* = 0.80), was analyzed.

The 113-item Youth Self Report (YSR; Achenbach & Rescorla, 2001) is parallel to the CBCL and TRF. The YSR is normed on a sample of 11–18-year-old youth (Achenbach & Rescorla, 2001). Although 154 youth in this study were 11 years or older at Wave 3, only 61 participants completed the measure due to time constraints. We used the age- and sex-adjusted Affective Problems T-score as another measure of self-reported depression. According to the manual, this subscale demonstrates satisfactory reliability (*α* = 0.81; Achenbach & Rescorla, 2001). All three variables for the proposed Wave 3 latent depression factor provided adjusted values; therefore, we utilized T-scores.

## Data Analytic Procedures

**Fig. 1:**
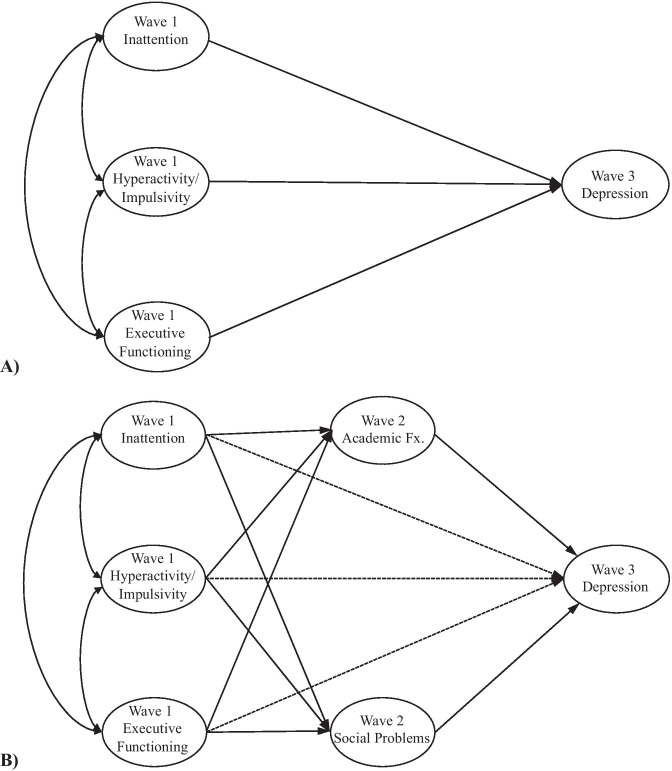
**A**) Proposed predictive model, testing the prospective prediction of Wave 3 self-reported depression from Wave 1 inattention, hyperactivity-impulsivity, and executive functioning. **B**) Proposed mediational model, testing Wave 2 academic functioning and social problems as mediators of the relationship between Wave 3 depression and baseline inattention, hyperactivity/impulsivity, and executive functioning. For **A** and **B**, circles indicate latent variables. Table 4 includes observed variables proposed to derive latent variables. Covariates are not included

We employed SEM to test Wave 1 ADHD (i.e., inattention, hyperactivity-impulsivity) and EF as predictors of Wave 3 depression (Fig. 1A). Next, we tested ADHD diagnostic status as a moderator to evaluate whether the prospective prediction of depression from EF differs according to ADHD diagnostic status. We finally examined academic functioning and social problems as mediators of the prospective prediction of Wave 3 depression from baseline inattention, hyperactivity-impulsivity, and EF (Fig. 1B). To test these proposed models, we implemented full information maximum likelihood (FIML; Enders, 2010), which performs well even with extreme missingness (e.g., 50%; Schlomer et al., 2010). We addressed FIML requirements that data are Missing at Random (MAR) or Missing Completely at Random (MCAR) and multivariate normality (Pritikin et al., 2018). Little’s Test suggested that the data violated the assumption of MCAR. Thus, we determined appropriate auxiliary variables to include to ensure data were missing at random and to improve power (Enders, 2010). Auxiliary variables are not central to the specific research question; rather, auxiliary variables may be highly correlated with missingness on the study variables or the included variables (Enders, 2010). We tested the correlation between potential auxiliary variables and variables relevant to the analyses as well as missingness on variables for the analyses. Several auxiliary variables significantly correlated with missingness and/or variables for analyses and were included in all SEM models (Table 2). For example, Wave 1 WIAT-II Math Reasoning standard score is not a variable in our analyses; however, it was determined to be an appropriate auxiliary variable because it was significantly correlated with: Wave 2 WIAT-II Word Reading standard score, Wave 2 WIAT-II Math Reasoning standard score, Wave 2 CBCL School Competence T-score missingness, Wave 2 CBCL Social Problems raw score missingness, and Wave 2 Parent Negative Social Preference missingness. For predictive models, Wave 2 academic and social mediators were implemented as auxiliary variables. Although Mardia’s test of Skewness [191.46, χ^2^(969) = 936.96, *p* = 0.76] did not violate the criteria for multivariate normality, Mardia’s test of Kurtosis [296.59, χ^2^ (1) = 7.02, *p* < 0.01] violated the assumption for the simplest model; therefore, we implemented maximum likelihood *robust* procedures in Mplus (Muthén & Muthén, 1998–2010) to address non-normality and accommodate missing data.

Prior to running SEM, we conducted separate confirmatory factor analyses on each Wave 1 (i.e., inattention, hyperactivity-impulsivity, EF), Wave 2 (i.e., academic functioning, social problems), and Wave 3 (i.e., depression) latent variables (see preliminary analyses). For proposed latent variables with three indicator variables (i.e., inattention, hyperactivity-impulsivity, EF, depression) we evaluated factor loadings to ensure that they exceeded the guidelines of at least 0.3 (Brown, 2014). For latent factors with four indicator variables (i.e., academic achievement, social problems), fit indices were examined. For predictive and mediational models, multiple fit indices were evaluated. A non-significant chi-square and comparative Fit Index (CFI) values ≥ 0.95 indicate good fit. Root mean square error of approximation (RMSEA) estimates model fit with control of sample size and per degrees of freedom where values ≤ 0.06 are acceptable (Hu & Bentler, 1999). Finally, a value of 0.08 or less for standardized root mean square residual (SRMR) suggests good model fit (Hu & Bentler, 1999).

Models conservatively accounted for age, sex, baseline depression, SES, and pubertal status. Age and sex were accounted for as the depression outcome observed variables adjusted for these factors. Additionally, we controlled for baseline age on the EF latent factor as EF improves with child development. Baseline depression, SES, and pubertal status were included as covariates. Consistent with other studies (e.g., Lawson & Farah, 2017), we utilized family income and parent education to approximate SES. To capture family income, we used a binary variable where $75,000 or less was coded as 0 and $75,001 or more was coded as 1 due to the fact that 66% of the sample with observed data on this variable had an income of more than $75,000. We also considered mother and father education in estimating SES. Education level was categorized as follows: 1 = eighth grade or less, 2 = some high school, 3 = high school graduate or GED, 4 = some college or post-high school, 5 = college graduate, 6 = advanced graduate or professional degree. The average value of mother and father education, which ranged from 1.5-6 (*M* = 4.92, *SD* = 0.91), was used; a single value from mother or father was utilized in circumstances when education data was present for one parent. Income and parent education variables were included in all SEM models. Children completed the CDI (Kovacs, 1992), as described above at baseline in addition to Wave 3. Each item is scored from 0 to 2, with 2 representing higher depression severity. The child age and sex adjusted T-score was used; norms for seven-year-olds were used to calculate T-scores for six-year-old children in the current sample. At Wave 3, children completed the Pubertal Development Scale (Petersen et al., 1988), a six-item self-report questionnaire used to assess pubertal status in males and females and noninvasively assesses pubertal status. Such measures are reliably associated with pubertal development as evaluated by the Tanner Scales (Carskadon & Acebo, 1993; Petersen et al., 1988) For the present study, a quantitative score described by Carskadon and Acebo (1993) was implemented. Each characteristic was rated from 1- 4 (1 = not yet started changing, 2 = has barely started changing, 3 = change is definitely underway, 4 = change seems completed) with the exception of a menstruation item for girls, which was coded as 1 for no and 4 for yes. The average of the items was used to measure puberty for the current study (*M* = 2.66, *SD* = 0.73). Finally, if inattention significantly predicted depression, we added anxiety as a covariate on the latent inattention factor to strengthen specific inferences (Pliszka, 2019). In addition to completing the CBCL at Wave 2 to assess youth academic and social functioning (see above for details), parents also completed this measure at baseline (i.e., Wave 1). The T-score from the Wave 1 Anxiety Problems subscale was used to assess child anxiety. Baseline anxiety was included as a covariate on the latent inattention variable in models where inattention emerged as a significant predictor.

## Results

### Preliminary Analyses

**Co-occurrence between ADHD and Depression.** To provide additional support for the use of dimensional conceptualization of ADHD, we tested for significant differences in Wave 1 and Wave 3 depression based upon baseline ADHD diagnostic status. Because considerable discrepancies exist between parent- and self-reported youth internalizing symptoms (De Los Reyes et al., 2015; Johnston & Murray, 2003; Lewis et al., 2014), youth self-reported depression measures were examined. On the CDI at baseline, 11 of 114 participants with complete data on this measure were at or above a T-score of 60 (i.e., high average and above). Seven of those 11 participants met diagnostic criteria for ADHD on the DISC-IV. This did not represent a significant difference in Wave 1 depression according to baseline ADHD status [χ^2^(1) = 1.08, *p* = 0.30]. At Wave 3, a total of 149 participating youth completed the CDI. Seven of those participants met or exceeded a T-score of 60, with four of those seven youth meeting diagnostic criteria for ADHD at Wave 1. Again, there was not a significant difference in Wave 3 depression on the CDI according to baseline ADHD diagnostic status [χ^2^(1) = 1.08, *p* = 0.30]. Similarly, there were no significant differences between Wave 3 depression and baseline ADHD diagnosis according to youth self-report on the RCADS depression subscale [χ^2^(1) = 1.82, *p* = 0.18]. Specifically, on the Wave 3 RCADS, two out of 148 youth met or exceeded the clinical cutoff (T-score above 70) for depression, both of whom had a diagnosis of ADHD at baseline. The fact that we did not observe a significant difference between those with and without ADHD at baseline and Wave 1 or Wave 3 depression demonstrates the importance of utilizing a dimensional conceptualization of ADHD when examining ADHD as a risk factor for depression.

**Confirmatory Factor Analyses.** Factor loadings from all confirmatory factor analyses are provided in Table 4. For the inattention, hyperactivity-impulsivity, and EF latent variables, standardized beta coefficients exceeded the recommended cutoff of 0.3 (Brown, 2014). The latent variables for Wave 2 academic functioning and social problems consisted of four observed variables each. The academic functioning factor demonstrated good fit across multiple indices (Table 4): χ^2^(2) = 4.42, *p* = 0.10, CFI = 0.98, and SRMR = 0.03, although the RMSEA value was 0.08. Because RMSEA cutoffs are vulnerable to poor fit in models with few degrees of freedom (Kenny et al., 2015), but other fit indices for this academic functioning latent variable were acceptable, a latent variable was implemented for academic functioning. In contrast, the social problems latent variable showed poor model fit [χ^2^(2) = 43.51, *p* < 0.001, CFI = .71, RMSEA = 0.33 and SRMR = 0.09]. Therefore, we created two composite variables to estimate parent- and teacher-rated social problems, respectively (see Table 3) by z-scoring the CBCL Social Problems raw score and the Dishion Negative Social Preference rating followed by calculating the average z-score; the same approach yielded a teacher-rated social functioning composite. Higher scores reflected worse social functioning.

### Prediction of Early Adolescent Depression

To review, controlling for baseline depression, SES, and pubertal status, we tested childhood inattention, hyperactivity-impulsivity, and EF as independent predictors of early adolescent depression; we also controlled for baseline age on the EF factor (depression outcomes were adjusted for age/grade and sex). When inattention significantly predicted depression, we also controlled for Wave 1 anxiety on inattention.

**Model 1.** We regressed a latent depression variable from Wave 3 on Wave 1 inattention, hyperactivity-impulsivity, and EF latent variables. Key indices suggested model misspecification [χ^2^(93) = 221.02, *p* < 0.001, CFI = 0.90, RMSEA = 0.08, SRMR = 0.08; Table 5]. Additionally, the latent variable covariance matrix was not positive definite, reflecting the high correlation between the inattention and hyperactivity-impulsivity latent variables. Wave 1 inattention positively predicted Wave 3 depression (β = 0.49, *SE* = 0.15, *p* = 0.001), but hyperactivity-impulsivity (β = -0.24, *SE* = 0.17, *p* = 0.14) and EF (β = 0.06, *SE* = 0.12, *p* = 0.60) did not. To further examine the initial aim of testing inattention, hyperactivity-impulsivity, and EF as prospective predictors of early adolescent depression, we modified the model as is recommended within SEM (Weston & Gore Jr., 2006; Violato & Hecker, 2007). Due to concerns regarding collinearity, we next tested a model evaluating total ADHD symptoms and EF as predictors of Wave 3 depression.

**Model 2.** We created three total ADHD symptom scores by summing the inattention and hyperactivity-impulsivity dimensions on the DISC-IV (*M* = 7.88, *SD* = 5.55), parent DBD (*M* = 20.46, *SD* = 14.00), and teacher DBD (*M* = 17.31, *SD* = 15.25). Confirmatory factor analyses revealed that these ADHD variables had acceptable factor loadings (> 0.49). We regressed the latent Wave 3 self-reported depression outcome on Wave 1 ADHD total symptom and EF latent variables including described covariates. This model improved fit (Table 5): χ^2^(59) = 78.70, *p* = 0.04, CFI = 0.97, RMSEA = 0.04, SRMR = 0.07. Although the chi-square was significant, this measure of fit is often unreliable (Vandenberg, 2006), so we prioritized other fit indices. For this alternative structural model, CFI, RMSEA, SRMR all demonstrated good fit where ADHD symptoms (β = 0.23, *SE* = 0.10, *p* = 0.01), but not EF (β = 0.02, *SE* = 0.13, *p* = 0.87), positively predicted depression. Model 2 accounted for 5.3% of the variance in Wave 3 depression.

**Model 3.** To improve specificity, we next tested EF and inattention as predictors of Wave 3 depression with identical covariates. The model fit the data well (Table 5) [χ^2^(59) = 82.79, *p* < 0.02, CFI = 0.96, RMSEA = 0.04, SRMR = 0.07] where inattention positively predicted Wave 3 early adolescent depression (β = 0.33, *SE* = 0.09, *p* < 0.001), but EF did not (β = 0.06, *SE* = 0.12, *p* = 0.60). In addition to demonstrating good fit to the data, Model 3 accounted for 9.0% of the variance in the Wave 3 latent depression variable.

**Model 4.** Hyperactivity-impulsivity and EF were also tested as predictors of depression. Although the model showed good fit (Table 5) [χ^2^(59) = 72.43 *p* < 0.11, CFI = 0.98, RMSEA = 0.03, SRMR = 0.07], it only accounted for 2.0% of the variance in Wave 3 depression. Additionally, neither hyperactivity-impulsivity (β = 0.10, *SE* = 0.11, *p* = 0.36) nor EF (β = -0.04, *SE* = 0.13 *p* = .77) predicted depression, suggesting overall that inattention is the primary risk factor for later depression.

**Fig. 2:**
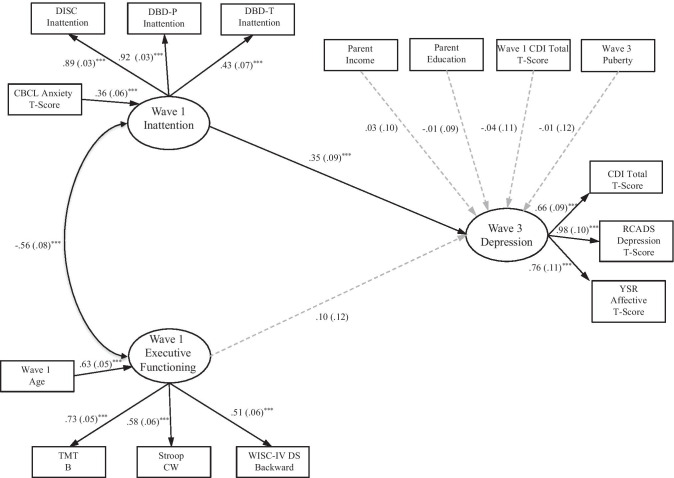
Represents Model 5 with standardized estimates. Standardized errors are in parentheses. Solid lines indicate significant relationships among variables. Inattention positively predicts early adolescent depression with control of SES, baseline depression, and puberty with additional control of anxiety on inattention. The model accounts for 10.8% of the variance in Wave 3 depression. ^***^p ≤ 0.001. ^**^p ≤ 0.01. ^*^p ≤ 0.05

**Model 5.** We reproduced Model 3 (i.e., inattention and EF predicting depression) but conservatively added baseline anxiety (i.e., CBCL Anxiety Problems). Even with control of anxiety on the latent inattention variable, the model showed good fit (Table 5) [χ^2^(72) = 99.09, *p* < 0.01, CFI = 0.96, RMSEA = 0.04, SRMR = 0.08] and accounted for 10.8% of the variance in Wave 3 depression. Baseline inattention continued to predict depression (β = 0.35, *SE* = 0.09, *p* < 0.001; Fig. 2) whereas EF did not (β = 0.10, *SE* = 0.12, p = 0.40).

**Multi-group Analysis.** We conducted multi-group analyses based on Wave 1 ADHD diagnostic status to determine whether predictions of youth depression from baseline EF differed according to group. However, this model had poor fit (SRMR = 0.22), suggesting that EF predicted depression similarly among youth with and without ADHD, though concerns related to power for multigroup models (Kline, 2015) limit conclusions.

### Early Adolescent Depression: Mediation by Academic Functioning and Social Problems

Using SEM, we tested academic functioning and social problems as temporally-ordered mediators of predictions of Wave 3 youth self-reported depression from baseline inattention, hyperactivity-impulsivity, and EF. Specifically, a Wave 2 latent academic functioning variable and two composite social problems variables (i.e., parent-report, teacher-report) were entered as mediators, controlling for the same covariates previously described.

A model consisting of Wave 2 academic functioning and social problems as mediators of predictions from Wave 1 latent inattention, hyperactivity-impulsivity, and EF showed poor fit [χ^2^(194) = 255.03 *p* < 0.01, CFI = 0.93, RMSEA = 0.04, SRMR = 0.09]. Examination of model pathways revealed no mediated effects. Wave 1 inattention inversely predicted Wave 2 academic functioning (β = -0.83, *SE* = 0.34, *p* = 0.01) and positively predicted Wave 2 parent-rated social problems (β = 0.35, *SE* = 0.17 *p* = 0.03). However, neither Wave 2 academic achievement (β = 0.20, *SE* = 0.23, *p* = 0.37) nor Wave 2 parent-rated social problems (β = 0.05, *SE* = 0.13, *p* = 0.71) predicted Wave 3 depression. Baseline hyperactivity-impulsivity and EF did not predict any mediators. Finally, none of the mediators (i.e., academic functioning, parent-rated social problems, teacher-rated social problems) predicted Wave 3 depression. There was a significant direct effect from Wave 1 inattention to Wave 3 depression (β = 0.68, *SE* = 0.30, *p* = 0.02), but no significant direct effects from hyperactivity-impulsivity (β = -0.44, *SE* = 0.24, *p* = 0.06) or EF (β = 0.00, *SE* = 0.15, *p* = 0.37) to Wave 3 depression emerged. Because no model pathways suggested significant mediation, respecified models were not investigated. We also tested a multigroup model with ADHD diagnostic status as a moderator of mediation by academic and social functioning, but the model’s failure to converge prevented strong inferences.

## Discussion

Identifying childhood predictors and mediators of depression, prior to the acute vulnerability in adolescence, will accelerate innovations in prevention and intervention. In a study of 216 youth ages 6–9, baseline inattention, hyperactivity-impulsivity, and EF were tested as simultaneous predictors of youth self-reported depression approximately four years later. Controlling for SES, baseline depression, and puberty as well as age (on the EF latent factor), inattention positively predicted early adolescent depression, even with added control of baseline anxiety in a model with inattention and EF as latent predictors. In contrast, academic functioning and social problems did not mediate predictions of depression from baseline inattention, hyperactivity-impulsivity, and EF.

The unique, robust association of early ADHD (inattention in particular) with early adolescent depression observed in this study is well-aligned with previous evidence, including predictions through young adulthood (Chronis-Tuscano et al., 2010; Meinzer et al., 2016). The centrality of inattention to depression was also reported previously, across multiple informants, in a cross-sectional study (Fenesy & Lee, 2019). Although inattention is transdiagnostic in nature, inattention assessed via measures of ADHD across informants (i.e., parent, teacher) prospectively predicted depression *independent* of co-occurring EF, anxiety, and baseline depression. Whereas youth with ADHD struggle with selective and sustained attention, anxiety is characterized by attentional biases secondary to threat (Weissman et al., 2012). Further, directed attention is generally a top-down cognitive process, but responses to threat are primarily mediated subcortically (Nigg, 2017). Thus, inattention secondary to ADHD is evident across settings and contexts and contributes to impairments across multiple domains, independently conferring risk for depression later in development. The hypothesis that symptoms of inattention and hyperactivity-impulsivity as well as EF deficits would predict multiple academic and social “failures” was not supported in this study. According to a competency-based model (Cole, 1991), self-esteem may be primary given its proximity to depression. Therefore, *interpretations or appraisals* of academic and social functioning may be more salient. For example, negative self-schema activated by stressors and/or low mood potentiates depression, including through maladaptive attributional styles (Jacobs et al., 2008). Given the centrality of multifinality to ADHD, across multiple settings and functional contexts, we await strong tests of cognitive factors (e.g., attributional style) and competency-based constructs, as mediators of depression. Further, because adolescence confers heighted sensitivity to social relationships, academic and social functioning measured at an older age may mediate emerging depression later in development from childhood ADHD (Powell et al., 2020). In the current study, social measures were not derived from self-report; instead, social functioning was estimated from broader constructs (i.e., social acceptance, social problems) according to informant report that may lack the precision necessary to capture successful peer interactions. For example, a multiple mediation model testing whether individual social skills (e.g., cooperation, empathy) collectively and uniquely mediate predictions of depression from inattention, hyperactivity-impulsivity, and EF could identify particular aspects of social behavior to target for intervention and reduce depression in youth. Social skills training improves social functioning and reduces the risk of depression in youth with autism spectrum disorder (Hotton & Coles, 2016), suggesting that continued research examining social functioning as an underlying mechanism of depression in youth with neurodevelopmental disorders is worthwhile and will facilitate innovations in intervention development. Overall, appropriate interventions targeting either a behavioral deficit or maladaptive cognitions could subsequently be applied to mitigate the risk of depression.

Critically, screening for childhood inattention may identify youth vulnerable to later onset depression. These individuals may benefit from prevention efforts (e.g., learning CBT skills to enhance mood) to mitigate depression onset and associated negative outcomes, in line with experimental evidence (Brent et al., 2015). Consistent with pediatric treatment guidelines, our findings emphasize the need for ongoing care and monitoring of children with ADHD, especially as inattentive symptoms are likely to persist into adolescence and adulthood (Wolraich et al., 2019) and may increase the likelihood of co-occurring depression. Effective intervention for childhood ADHD requires treatment by a multidisciplinary team to properly assess and intervene prior to depression onset (e.g., medical providers, psychologists, educators; Barbaresi, 2020). Future studies should test the associations observed in the present study in youth diagnosed with major depressive disorder or those at elevated risk (e.g., offspring of depressed mothers) as well. Parental depression, for example, is a robust risk factor for adolescent depression. If inattention symptoms predicted clinically significant depression in other populations, this would substantiate the rationale to screen inattention even in the absence of ADHD. Additionally, greater refinement of specific inattention symptoms or combinations of symptoms that increase risk for later depression would enable clinicians to improve screening techniques.

Surprisingly, with control of baseline ADHD, childhood EF did not predict early adolescent depression. Consistent with evidence that EF impairments remit following a depressive episode in adults and adolescents (Biringer et al., 2005; Maalouf et al., 2011), present findings suggest that childhood EF did not uniquely confer vulnerability for early adolescent depression. However, developmentally-sensitive socio-emotional and neurobiological changes critically contextualize these findings. For example, most of the current participants had only begun their transition to adolescence, thus still undergoing well-characterized developmental unfolding of limbic systems and prefrontal regions underlying emotion dysregulation and heightened reward sensitivity (Powers & Casey, 2015). Further, poor EF may be more acutely related to adolescent-onset depression because frontal networks supporting cognitive down-regulation of negative emotionality are still emerging. EF measures continue to suffer from poor ecological validity whereas rating scales may prove more useful (Barkley & Murphy, 2011). Similarly, computerized EF measures, which include more accurate assessments of subtle variations in response time (e.g., NIH ToolBox; Zelazo et al., 2014), may show better predictive properties. Finally, we used a latent EF variable, derived from multiple EF domains, to reduce measurement error. However, differentiated measures of EF may yield more specific patterns of association. For example, better inhibition in adults supported reappraisal of negative stimuli and effective emotion suppression that mitigated depressive symptoms (Joormann & Gotlib, 2010).

The present study had several key strengths and limitations. First, the SEM approach reduced measurement error and Type I error, which is particularly important for EF given its multidimensionality. We also utilized temporally-ordered, multi-method/informant data to test causal mediation. Important limitations include a sample recruited for ADHD at baseline, but one not specifically designed to capture youth depression. Thus, few participants demonstrated clinically significant depression in early adolescence. At Wave 3, only one participant had completed pubertal development, a key limiting factor given the dramatic increase in psychopathology secondary to pubertal timing. These aspects of the study design, in combination with the prospective longitudinal framework, likely contributed to the somewhat low variance accounted for the Wave 3 latent depression variable. Variance accounted for would likely increase if replicated in samples studying post-pubertal adolescents and if other risk factors highly predictive of youth depression (e.g., maternal depression; Hammen & Brennan, 2003) were included. Nevertheless, the fact that childhood inattention significantly predicted early adolescent depression approximately four years later remains an important finding and may be particularly critical to study in a sample of youth who all meet diagnostic criteria for ADHD. With respect to multigroup models, the present study may have been underpowered to detect effects given 216 participants, missing data, and inclusion of several parameters (Kline, 2015); therefore, results related to the multigroup model must be interpreted with caution. Multigroup models examining whether the prospective prediction of depression from childhood EF varies according to ADHD diagnostic status should be evaluated in larger samples to improve confidence in our results. Additionally, consideration of sex and age moderators would be compelling, especially as the risk for depression increases during adolescence and the prevalence of depression is higher in adolescent girls relative to boys (Thapar et al., 2012). Finally, a small number of participants with a diagnosis of ADHD were on stimulant medication on the day of baseline testing (n = 13). Simulant medication improves performance on some EF dimensions in children with ADHD (Barnett et al., 2001; Snyder et al., 2008). Medication use in ADHD is likely most reflective of disorder severity due to the intervention selection bias, and because the current study did not use random assignment we are not positioned to make inferences about treatment effects (Larzelere et al., 2004).

We tested the prospective association of childhood ADHD and EF with depression in early adolescence, including mediation by academic functioning and social problems. Latent inattention positively predicted youth self-reported depression, but hyperactivity-impulsivity and EF did not. These findings support the centrality of inattention as a unique risk factor for depression, demonstrating the importance of screening for inattention and interventions to improve inattention and reduce risk. Continued investigation of underlying mechanisms between inattention and early adolescent depression will provide additional targets for intervention.

**Table 1:** Descriptive Statistics of Demographics and Key Study Variables

*Variable*	*M (SD) or % of Sample*	*Range*	*n*
Wave 1 Age	7.39 (1.07)	6-9	216
Wave 2 Age	9.68 (1.27)	7-13	193
Wave 3 Age	12.07 (1.30)	9-15	172
Sex (% Male)	66.67	-	216
Race-Ethnicity (% Caucasian)	50.93	-	216
SES			
Income (% $75,001 or more)	65.99	-	197
Parent Education	4.92 (0.91)	1.5-6	202
Wave 1 Depression (CDI T-Score)	46.84 (7.47)	35-71	144
Wave 3 Puberty	2.27 (0.73)	1-3.8	143
Wave 1 Anxiety (CBCL Anxiety Problems T-Score)	56.21 (7.49)	(50-75)	214
FSIQ	107.29 (14.24)	73-144	216
Wave 1 Inattention Symptoms			
Inattention Symptoms (DSIC-IV)	4.54 (3.16)	0-9	216
Inattention Symptoms (DBD Parent)	11.17 (7.63)	0-27	210
Inattention Symptoms (DBD Teacher)	9.35 (8.33)	0-27	150
Wave 1 Hyperactivity-Impulsivity Symptoms			
Hyperactivity-Impulsivity Symptoms (DSIC-IV)	3.34 (3.08)	0-9	216
Hyperactivity-Impulsivity Symptoms (DBD Parent)	9.32 (7.33)	0-27	209
Hyperactivity-Impulsivity Symptoms (DBD Teacher)	7.79 (8.38)	0-27	150
Wave 1 Executive Functioning			
TMT-B (min)	-1.22 (0.81)	-5.02 - -0.27	212
WISC-IV DSB Raw	5.86 (1.58)	0-10	210
Stroop C-W	21.42 (6.43)	4-41	201
Wave 2 Academic Functioning			
WIAT-II Word Reading Standard Score	107.58 (14.27)	53-141	184
WIAT-II Math Reasoning Standard Score	111.86 (16.82)	61-160	183
School Competence T-Score (CBCL)	44.79 (9.46)	24-55	182
Academic Performance T-Score (TRF)	49.27 (10.12)	35-65	95
Wave 2 Social Problems			
Negative Social Preference (Parent Dishion)	-2.60 (1.85)	-4-4	172
Negative Social Preference (Teacher Dishion)	-2.18 (2.13)	-4-4	92
Social Problems Raw (CBCL)	3.27 (3.48)	0-16	188
Social Problems Raw (TRF)	2.28 (2.67)	0-14	91
Wave 3 Depression			
CDI T-Score	44.14 (7.74)	34-75	149
RCADS T-Score	43.91 (10.55)	30-78	148
Affective Problems T-Score (YSR)	54.66 (7.49)	50-80	61

*SES* = Socioeconomic Status, *CDI* = Children's Depression Inventory, *CBCL* = Child Behavior Checklist, *DISC-IV* = Diagnostic Interview Schedule for Children Fourth Edition, *DBD* = Disruptive Behavior Disorder Rating Scale, *TMT-B* = Trail Making Test Part B, *WISC-IV* = Wechsler Intelligence Scale for Children-Fourth Edition, *DSB* = Digit Span Backwards, Stroop *C-W* = Stroop Color-Word Condition, *WIAT-II* = Wechsler Individual Achievement Scale Second Edition, *TRF* = Teacher Report Form, *RCADS* = Revised Children's Anxiety and Depression Scale, *YSR* = Youth Self-Report

**Table 2:** Auxiliary Variables

**Demographic**	**Wave 1**	**Wave 2**	**Wave 3**
Child Age (Waves 2 and 3)	WIAT-II Word Reading SS	DISC-IV Inattention Symptoms	CDI Total Raw Score
Child Sex	WIAT-II Math Reasoning SS	DISC-IV HI Symptoms	RCADS Depression Raw Score
FSIQ	CBCL School Competence T-score	DBD-Parent Inattention Symptoms	YSR Affective Problems Raw Score
	CBCL Social Problems T-score	DBD-Parent HI Symptoms	
	TRF Academic Performance T-score	DBD-Teacher Inattention Symptoms	
	TRF Social Problems T-score	DBD-Teacher HI Symptoms	
		TMT-B	
		Stroop C-W Condition	
		WISC-IV Digit Span Backwards	
		RCADS Depression T-score	

*FSIQ* = Full Scale IQ, *WIAT-II*  = Wechsler Individual Achievement Scale Second Edition; *SS* = Standard Score, *CBCL* = Child Behavior Checklist, *TRF* = Teacher Report Form, *DISC-IV* = Diagnostic Interview Schedule for Children Fourth Edition, *HI = *Hyperactivity-Impulsivity, *DBD* = Disruptive Behavior Disorder Rating Scale; *TMT-B* = Trail Making Test Part B, *Stroop C-W = *Stroop Color-Word Condition, *WISC-IV* = Wechsler Intelligence Scale for Children-Fourth Edition, *RCADS* = Revised Children's Anxiety and Depression Scale, *CDI = *Children's Depression Inventory, *YSR = *Youth Self-Report

**Table 3:** Correlations Among Covariates, Predictor, Mediator, and Outcome Variables

Variable	1	2	3	4	5	6	7	8	9	10	11	12	13
1. Wave 1 Age	-												
2. Sex (% Male)	-0.09	-											
3. Income (1 = $75,001 or more)	-0.05	0.12	-										
4. Parent Education	-0.04	0.14^*^	0.46^***^	-									
5. Wave 1 Depression (CDI T-Score)	0.11	-0.17^*^	-0.27^***^	-0.32^***^	-								
6. Wave 3 Puberty	0.49^***^	-0.35^***^	-0.07	-0.02	0.17	-							
7. Wave 1 Anxiety (CBCL Anxiety Problems T-Score)	0.04	0.07	-0.03	-0.03	0.06	0.06	-						
8. FSIQ	-0.04	0.13^*^	0.15^*^	0.35^***^	-0.17^*^	-0.07	-0.06	-					
9. Wave 1 Inattention Symptoms (DSIC-IV)	-0.03	0.11	-0.09	-0.08	0.24^**^	0.01	0.34^***^	-0.24^***^	-				
10. Wave 1 Inattention Symptoms (DBD P)	-0.05	0.09	-0.06	-0.09	0.17^*^	0.03	0.31^***^	-0.26^***^	0.82^***^	-			
11. Wave 1 Inattention Symptoms (DBD T)	-0.10	0.15	-0.11	-0.04	0.14	0.06	0.08	-0.28^***^	0.40^***^	0.41^***^	-		
12. Wave 1 Hyperactivity-Impulsivity Symptoms (DSIC-IV)	-0.19^**^	0.19^**^	0.10	-0.04	0.10	0.02	0.32^***^	-0.03	0.58^***^	0.60^***^	0.28^***^	-	
13. Wave 1 Hyperactivity-Impulsivity Symptoms (DBD P)	-0.16^*^	0.16^**^	0.10	-.04	0.09	0.05	0.34^***^	-0.08	0.61^***^	0.75^***^	0.34^***^	0.87^***^	-
14. Wave 1 Hyperactivity-Impulsivity Symptoms (DBD T)	-0.31^***^	0.21^**^	0.10	-0.03	0.11	0.14	0.14	-0.14	0.21^**^	0.28^***^	0.64^***^	0.49^***^	0.49^***^
15. Wave 1 TMT-B (min)	0.46^***^	0.00	-0.02	0.05	-0.01	0.18^*^	0.06	0.38^***^	-0.27^***^	-0.28^***^	-0.15	-0.19^**^	-0.18^**^
16. Wave 1 WISC-IV DSB Raw	0.29^***^	0.02	0.08	0.10	-0.11	0.09	0.02	0.38^***^	-0.12	-0.15^*^	-0.12	-0.14^*^	-0.09
17. Wave 1 Stroop C-W	0.38^***^	-0.04	0.09	0.06	-0.06	0.22^**^	-0.04	0.31^***^	-0.23^***^	-0.27^***^	-0.23^**^	-0.15^*^	-0.16^*^
18. Wave 2 WIAT-II Word Reading Standard Score	-0.08	0.05	0.14	0.16^*^	-0.21^**^	-0.08	0.04	0.57^***^	-0.24^***^	-0.24^***^	-0.31^***^	-0.06	-0.11
19. Wave 2 WIAT-II Math Reasoning Standard Score	-0.10	0 .11	0.21^**^	0.39^***^	-0.16	-0.06	-0.07	0.76^***^	-0.31^***^	-0.36^***^	-0.31^***^	-0.14^*^	-0.18^**^
20. Wave 2 School Competence T-Score (CBCL)	-0.02	0.07	0.27^***^	0.28^***^	-0.21^**^	0.01	-0.21^**^	0.59^***^	-0.48^***^	-0.49^***^	-0.38^***^	-0.24^***^	0.33^***^
21. Wave 2 Academic Performance T-Score (TRF)	0.08	0.05	0.28^**^	0.26^**^	-0.26^*^	0.16	-0.02	0.55^***^	-0.31^**^	-0.32^**^	-0.39^***^	-0.04	-0.07
22. Wave 2 Negative Social Preference (Parent Dishion)	0.07	0.01	-0.00	-0.07	0.10	0.08	0.26^***^	-0.16^*^	0.35^***^	0.38^***^	0.28^***^	0.32^***^	0.37^***^
23. Wave 2 Negative Social Preference (Teacher Dishion)	0.08	0.13	-0.13	-0.08	0.12	0.04	0.31^**^	-0.16	0.22^*^	0.20	0.28^*^	0.23^*^	0.23^*^
24. Wave 2 Social Problems Raw (CBCL)	-0.01	0.01	-0.02	-0.16^*^	0.10	0.03	0.39^***^	-0.24^***^	0.41^***^	0.46^***^	0.23^*^	0.46^***^	0.49^***^
25. Wave 2 Social Problems Raw (TRF)	0.02	0.13	-0.03	-0.03	0.01	0.00	0.19^*^	-0.13	0.22^*^	0.25^**^	0.07	0.26^***^	0.25^**^
26. Wave 3 CDI T-Score	0.17^*^	-0.23^**^	-0.13	-0.15	0.09	0.28^***^	0.27^***^	-0.17^*^	0.11	0.17^*^	0.22^*^	-0.02	-0.00
27. Wave 3 RCADS T-Score	0.06	0.03	-0.02	-0.06	0.00	0.03	0.32^***^	-0.17^*^	0.24^**^	0.28^***^	0.12	0.10	0.14
28. Wave 3 Affective Problems T-Score (YSR)	0.04	0.07	0.02	0.09	0.23	0.17	0.14	0.13	0.07	0.14	0.05	0.13	0.11

Variable	14	15	16	17	18	19	20	21	22	23	24	25	26	27
14. Wave 1 Hyperactivity-Impulsivity Symptoms (DBDT)	-													
15. Wave 1 TMT-B (min)	-0.14	-												
16. Wave 1 WISC-IV DSB Raw	-0.13	0.45^***^	-											
17. Wave 1 Stroop C-W	-0.11	0.34^***^	0.26^***^	-										
18. Wave 2 WIAT-II Word Reading Standard Score	-0.17^*^	0.25^***^	0.36^***^	0.20^**^	-									
19. Wave 2 WIAT-II Math Reasoning Standard Score	-0.11	0.39^***^	0.39^***^	0.26^***^	0.61^***^	-								
20. Wave 2 School Competence T-Score (CBCL)	-0.22^**^	0.33^***^	0.25^***^	0.26^***^	0.56***	0.60^***^	-							
21. Wave 2 Academic Performance T-Score (TRF)	-0.07	0.30^**^	0.38^***^	0.28^**^	0.50^***^	0.57^***^	0.68^***^	-						
22. Wave 2 Negative Social Preference (Parent Dishion)	0.25^**^	-0.10	-0.03	-0.19^**^	-0.12	-0.15^*^	-0.38^***^	-0.18	-					
23. Wave 2 Negative Social Preference (Teacher Dishion)	0.26^*^	-0.01	0.02	0.04	-0.04	-0.25^**^	-0.34^***^	-0.28^**^	0.54***^***^	-				
24. Wave 2 Social Problems Raw (CBCL)	0.23^**^	-0.18^**^	-0.13	-0.18^**^	-0.17^*^	-0.31^***^	-0.45^***^	-0.21^*^	0.67^***^	0.37^***^	-			
25. Wave 2 Social Problems Raw (TRF)	0.21	-0.23^*^	0.03	-0.04	-0.12	-0.25^**^	-0.33^***^	-0.25^**^	0.45^***^	0.71^***^	0.38^***^	-		
26. Wave 3 CDI T-Score	-0.01	0.01	-0.00	0.05	-0.10	-0.15	-0.21^**^	-0.15	0.18^*^	0.16	0.18^*^	0.06	-	
27. Wave 3 RCADS T-Score	0.00	-0.10	-0.02	-0.09	-0.03	-0.12	-0.13	-0.07	0.23^**^	0.20	0.07	0.16	0.64^***^	-
28. Wave 3 Affective Problems T-Score (YSR)	0.06	0.13	0.22	0.17	0.21	0.11	-0.02	0.13	0.06	0.12	-0.09	-0.07	0.57^***^	0.76^***^

*CDI* = Children's Depression Inventory, *CBCL* = Child Behavior Checklist, *DISC-IV* = Diagnostic Interview Schedule for Children Fourth Edition, *DBD* *P* = Disruptive Behavior Disorder Rating Scale Parent Report, *DBD* *T* = Disruptive Behavior Disorder Rating Scale Teacher Report, *TMT-B* = Trail Making Test Part B, *WISC-IV* = Wechsler Intelligence Scale for Children-Fourth Edition, *DSB = *Digit Span Backwards; *Stroop C-W* = Stroop Color-Word Condition, *WIAT-II* = Wechsler Individual Achievement Scale Second Edition, *TRF* = Teacher Report Form; *RCADS* = Revised Children's Anxiety and Depression Scale, *YSR = *Youth Self-Report

***p ≤ 0.001; **p ≤ 0.01; *p ≤ 0.05

**Table 4:** Factor Loadings for Confirmatory Factor Analyses

*Factor*	*Factor Loading*	*SE*	*z*	*p*
Wave 1 Inattention*				
Inattention Symptoms (DSIC-IV)	0.85	0.06	13.29	<0.001
Inattention Symptoms (DBD Parent)	0.96	0.06	15.40	<0.001
Inattention Symptoms (DBD Teacher)	0.44	0.07	6.07	<0.001
Wave 1 Hyperactivity-Impulsivity*				
Hyperactivity-Impulsivity Symptoms (DSIC-IV)	0.93	0.04	21.92	<0.001
Hyperactivity-Impulsivity Symptoms (DBD Parent)	0.93	0.04	23.78	<0.001
Hyperactivity-Impulsivity Symptoms (DBD Teacher)	0.53	0.07	14.95	<0.001
Wave 1 Executive Functioning*				
TMT-B (min)	0.76	0.08	9.39	<0.001
WISC-IV DSB Raw	0.58	0.07	7.36	<0.001
Stroop C-W	0.48	0.07	6.48	<0.001
Wave 2 Academic Functioning*				
WIAT-II Word Reading Standard Score	0.73	0.05	16.06	<0.001
WIAT-II Math Reasoning Standard Score	0.77	0.05	16.21	<0.001
School Competence T-Score (CBCL)	0.77	0.05	15.66	<0.001
Academic Performance T-Score (TRF)	0.81	0.06	14.61	<0.001
**Wave 2 Social Problems**				
Negative Social Preference (Parent Dishion)	0.85	0.07	12.26	<0.001
Negative Social Preference (Teacher Dishion)	0.67	0.12	5.67	<0.001
Social Problems Raw (CBCL)	0.74	0.06	11.82	<0.001
Social Problems Raw (TRF)	0.64	0.11	5.62	<0.001
Wave 3 Depression*				
CDI T-Score	0.70	0.05	13.39	<0.001
RCADS T-Score	0.92	0.06	14.90	<0.001
Affective Problems T-Score (YSR)	0.79	0.07	10.68	<0.001

*DISC-IV* = Diagnostic Interview Schedule for Children Fourth Edition; *DBD* = Disruptive Behavior Disorder Rating Scale, *TMT-B* = Trail Making Test Part B, *WISC-IV* = Wechsler Intelligence Scale for Children-Fourth Edition, *DSB* = Digit Span Backwards, *Stroop C-W* = Stroop Color-Word Condition, *WIAT-II* = Wechsler Individual Achievement Scale Second Edition, *CBCL* = Child Behavior Checklist, *TRF* =  Teacher Report Form, *CDI = *Children's Depression Inventory, *RCADS* = Revised Children's Anxiety and Depression Scale, *YSR* = Youth Self-Report

*Indicates Latent Factors Used in Analyses

**Table 5:** Predictive Model Fit Indices

*Model*	χ2	*CFI*	*RMSEA*	*SRMR*
Model 1 (Inattention, Hyperactivity-Impulsivity, EF)	χ^2^(93) = 221.02, *p*< 0.001	**0.90**	**0.08**	**0.08**
Model 2 (ADHD, EF)	χ^2^(59) = 78.70, *p*= 0.04	**0.97**	**0.04**	**0.07**
Model 3 (Inattention, EF)	χ^2^(59) = 82.79, *p*< 0.02	**0.96**	**0.04**	**0.07**
Model 4 (Hyperactivity-Impulsivity, EF)	**χ2(59) = 72.43** ***p*< 0.11**	**0.98**	**0.03**	**0.07**
Model 5 (Inattention with control of anxiety, EF)	χ^2^(72) = 99.09, *p*< 0.01	**0.96**	**0.04**	**0.08**

Latent predictor variables in parentheses. Bold text indicates good model fit based on the index
